# The relationship between medical staff burnout and subjective wellbeing: the chain mediating role of psychological capital and perceived social support

**DOI:** 10.3389/fpubh.2024.1408006

**Published:** 2024-06-21

**Authors:** Jia Fan, Yuyang Chang, Li Li, Nan Jiang, Zhifei Qu, Jiaxin Zhang, Meihua Li, Bing Liang, Danhua Qu

**Affiliations:** ^1^The Second Hospital of Jilin University, Jilin, China; ^2^School of Nursing, Jilin University, Jilin, China; ^3^National Center for Children’s Health, Beijing Children’s Hospital, Capital Medical University, Beijing, China

**Keywords:** medical staff, burnout, subjective wellbeing, psychological capital, perceived social support

## Abstract

**Background:**

Medical staff play a crucial role in delivering healthcare services, especially during epidemics of infectious diseases such as coronavirus disease 2019 (COVID-19). However, there is a growing issue of burnout and low wellbeing among this group. While it is widely recognized that burnout has a negative impact on subjective wellbeing, the exact relationship between the two is not yet completely understood. The purpose of this study is to explore the chain mediating role of psychological capital and perceived social support between burnout and subjective wellbeing among medical staff.

**Methods:**

Using the convenient sampling method, 604 medical staff were selected for a cross-sectional study. All participants completed a self-report questionnaire that collected demographic information, as well as data from the Maslach Burnout Inventory-Human Services Survey, General Wellbeing Schedule, Psychological Capital Questionnaire, and Perceived Social Support Scale. SPSS 27.0 and SPSS PROCESS macro were used for data analysis.

**Results:**

There was a significant correlation between burnout, psychological capital, perceived social support, and subjective wellbeing (*p* < 0.01). Burnout not only has a direct negative impact on the subjective wellbeing of medical staff (effect: −0.2045; Bootstrap 95%CI: −0.2506, −0.1583), but also exerts an indirect influence on subjective wellbeing through three pathways: the independent mediating effect of psychological capital (effect: −0.0481; Bootstrap 95%CI: −0.0876, −0.0109), the independent mediating effect of perceived social support (effect: −0.0092; Bootstrap 95%CI: −0.0203, −0.0003), and the chained mediating effect of psychological capital and perceived social support (effect: −0.0092; Bootstrap 95%CI: −0.0183, −0.0019).

**Conclusion:**

High burnout in medical staff can impair the level of psychological capital, leading to diminished perceived social support and ultimately reduced subjective wellbeing. The findings of this study contribute to understanding the potential pathways between burnout and subjective wellbeing and provide preliminary data support for developing strategies to improve the mental health of medical staff.

## 1 Background

With the sudden emergence of the coronavirus disease 2019 (COVID-19) pandemic and its continuously evolving variants posing significant challenges to the healthcare system, along with the advancements in the increasingly complex medical environment today, there are progressively higher demands placed on medical staff (1, 2). It is emphasized as a necessary condition to achieve universal health coverage to have a competent and qualified healthcare workforce to address epidemiological challenges and evolving health needs (3). However, there is a global shortage of medical staff (4, 5), leading to heavy workloads and decreased focus on the job. This can easily jeopardize physical and mental health, create burnout, and cause a decline in wellbeing.

Positive psychology is a pioneering method for dealing with burnout and enhancing wellbeing. It utilizes individuals’ potential, strengths, and functions to foster personal thriver (6, 7). In the field of positive psychology, the construction of wellbeing is attributed to the first pillar (i.e., positive subjective experiences), and subjective (or “hedonic”) wellbeing (SWB) is one of its important concepts (6). It pertains to how individuals assess the overall quality of their lives based on their criteria. It is widely used as a measure to evaluate mental health (8). This encompasses both reflective cognitive judgments (e.g., life satisfaction) and emotional reactions to one’s present circumstances (i.e., pleasant and unpleasant emotions) (9). SWB has gradually been recognized as a symbol of national prosperity (10). Since the introduction of positive psychology, research on individuals’ SWB has become increasingly popular, especially within specific organizational contexts (11). Medical staff are responsible for saving lives and caring for patients, serving as the backbone of healthcare services. Given the prominence of the healthcare system in social services, ensuring the quality of the work life of medical staff is an important factor in maintaining its stability. Medical staff’s sense of achievement and satisfaction in their professional lives has been conceptualized as occupational wellbeing (12), which is closely related to broader psychological wellbeing or SWB (13). Therefore, exploring the factors influencing the SWB of medical staff is essential for improving their SWB and further enhancing the level of medical care. To date, a large number of studies at home and abroad have explored the influencing factors of SWB (14–16).

Among the many factors that influence SWB, burnout is an important variable. The World Health Organization (WHO) defines burnout as an “occupational phenomenon” in the International Classification of Diseases 11th revision (17, 18). It is a syndrome caused by chronic work stress that has not been successfully managed and is a serious health problem (17, 18). It has three characteristics: feelings of energy depletion or exhaustion; increased psychological distance from one’s job, feelings of negativism or cynicism related to one’s job; and feelings of ineffectiveness and lack of accomplishment (17, 18). Studies show that burnout is common among medical staff, and this is a global phenomenon (19–22). During the COVID-19 pandemic, studies show that around two-thirds of medical staff experience high levels of burnout, which has become a crucial issue to consider (23, 24). According to a nationwide cross-sectional survey, the prevalence of burnout among Chinese medical staff is 60.8% (25), far higher than the global prevalence (21). The high incidence of medical staff burnout is directly reflected in personal physical health (26) (e.g., alcohol abuse or alcohol dependence), and work level (27) (e.g., negative work attitude, turnover intention). This will directly lead to a decline in the quality of patient care and an increase in medical malpractice litigation, resulting in direct economic costs and indirect reputation costs (28, 29). Not only that, but burnout has become one of the important factors hindering SWB (11), and it has a significant negative predictive effect on SWB (30–32). However, the potential impact path between burnout and SWB is not yet completely clear. Some studies have found that burnout has a predictive effect on mental health, and positive mental health can reduce burnout (27, 33). Therefore, it is necessary to understand the mechanisms, whether internal and external factors such as psychological capital (PsyCap) and social support are involved as mediators, to take effective measures based on positive psychology to prevent and reduce burnout and increase SWB.

The second pillar of positive psychology (i.e., positive individual traits) proposes that character strengths can enrich and promote various aspects of wellbeing (6, 34). PsyCap, as an extension of positive psychology ideas in the field of organizational behavior, refers to the positive psychological state or mental energy of an individual in the process of development or growth and contains four core components: self-efficacy, optimism, hope, and resilience (35). Specifically, it fosters confidence to succeed in the face of challenging tasks (self-efficacy), optimism about current and future success (optimism), and adherence to a positive motivational state that can re-establish pathways to achieve goals when needed (hope) (36). In addition, PsyCap provides a flexible ability to recover from failure, adversity, uncertainty, or overwhelming change (resilience) (36). Theoretical and practical evidence demonstrates that the malleability of PsyCap implies that its four components are sustainable and can have a wide-ranging impact on individuals’ attitudes and behaviors (37). For example, PsyCap can improve nursing burnout (38), alleviate depression (39), and influence wellbeing (40). Existing research has shown that PsyCap of medical staff has a significant negative impact on burnout (41–43). Furthermore, PsyCap plays a mediating role in the outcomes of nursing staff burnout, such as nursing performance (44). Medical staff’s PsyCap positively predicts occupational wellbeing (40). However, few studies have considered how burnout, PsyCap, and SWB are interrelated and interacted with each other. Therefore, it suggests that PsyCap can not only directly negatively impact medical staff’s burnout and positively predict SWB, but also serve as a mediating variable among relevant factors. Based on this, this study proposes Hypothesis 1 (H1): burnout can affect SWB through the mediating effect of PsyCap.

A positive social environment is the third pillar of positive psychology, manifested as positive groups and social institutions that are important for individual health (6). Social support as a powerful external factor refers to experiences valued, nurtured, and respected by those closest to the individual, who may receive support from various sources, including people and society. Perceived social support (PSS) refers to the degree to which individuals feel subjectively understood, respected, and supported by others in the social environment. It emphasizes individuals’ subjective perception, experience, and understanding of different types of social support, and influences their psychological wellbeing more than the actual social support (45). Research has found that through the help and protection provided by others, medical staff can to some extent avoid the occurrence of burnout (46, 47). Higher PSS is associated with lower burnout (48), higher wellbeing (49), higher PsyCap (43), and lower psychological distress (50). A review conducted among workers during the COVID-19 pandemic found a correlation between organizational support and the mental health of this population (51). The investigation of female physicians in Yantai City, Shandong Province, China, indicates that perceived family support plays a moderating role between burnout and SWB (49). Although previous studies have suggested the connection between PSS, burnout, and SWB among medical staff, there is still a need for further exploration due to the limited sample size and narrow research focus. Therefore, we propose Hypothesis 2 (H2): burnout can affect SWB through the mediating effect of PSS. In addition, considering the significant correlation between PSS and PsyCap among medical staff (43), as well as the positive predictive role of PsyCap on PSS in volunteers (52). Our Hypothesis 3 (H3) is that among medical staff, burnout can affect SWB through the chain intermediary effect of PsyCap and PSS.

**Figure 1:**
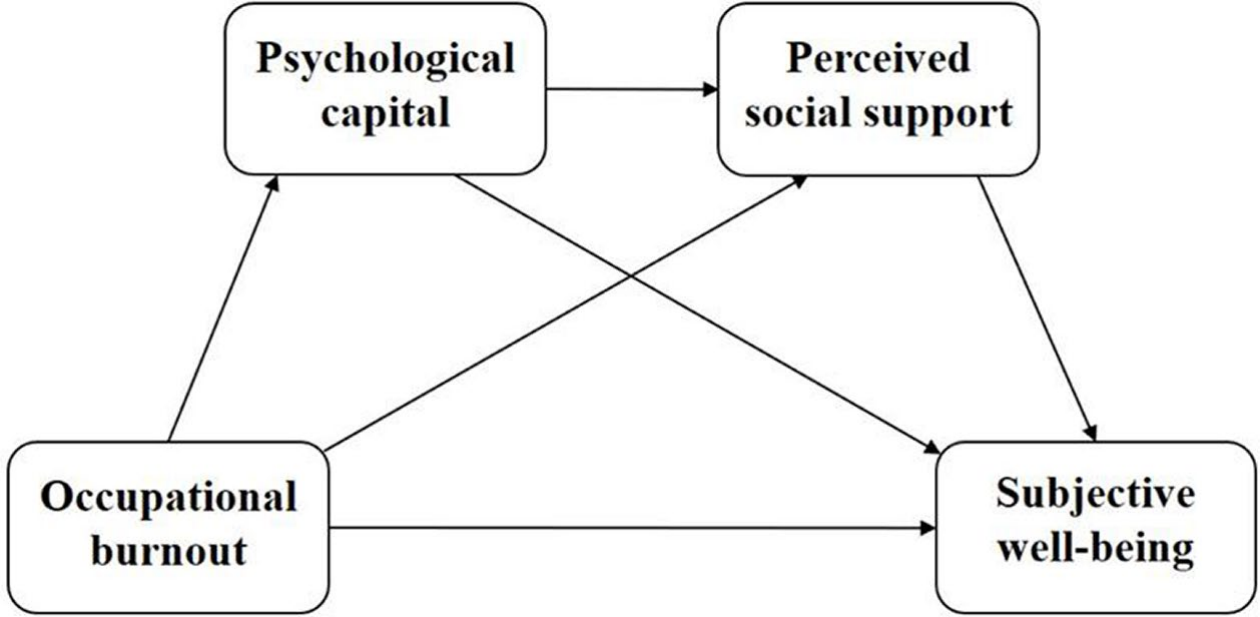
Hypothesis model.

Previous research on medical staff burnout has focused on negative aspects (e.g., stress, depression), with less research on positive psychological emotion aspects. At the same time, despite previous research indicating the association between physician or nurse burnout, SWB, PsyCap, and PSS, in the actual clinical environment, physicians, technicians, and nurses in various departments do not exist independently but rather act as a unified whole in a division of labor and cooperation to play a therapeutic role. Currently, there is still a lack of in-depth exploration of the intrinsic structural relationships between these variables in the real clinical environment. Given the high prevalence of burnout among medical staff and the importance of SWB, exploring potential pathways between the two will contribute to a thriving medical workforce. In summary, this study has established a hypothetical model (Figure 1) aimed at clarifying the pathways and impact of the roles of medical staff burnout, SWB, PsyCap, and PSS, to provide a certain reference for future exploration of pathways to reduce medical staff burnout and enhance wellbeing.

## 2 Methods

### 2.1 Study design and participants

A cross-sectional study was conducted using a convenient sampling method from March to June 2022, involving 631 medical staff. The study followed the Strengthening the Reporting of Observational Studies in Epidemiology (STROBE) checklist (53) (Supplementary Table S1). Participants were involved in the treatment or support work of the largest designated hospital for epidemic prevention and control in Changchun, China. Their original working units were, respectively, the First Hospital of Jilin University, the Second Hospital of Jilin University, the China-Japan Union Hospital of Jilin University, the Jilin Province People’s Hospital, the Affiliated Hospital of Changchun University of Traditional Chinese Medicine, and the Changchun Hospital of Traditional Chinese Medicine, a total of 6 tertiary medical institutions. They were invited to complete an online survey on the Questionnaire Star platform.^1^ The survey included self-reported questionnaires on demographic information, burnout, SWB, PsyCap, and PSS. The inclusion criteria were: (1) Medical staff who have worked in designated hospitals for infectious diseases including COVID-19 and were involved in treatment or support work for a minimum of 1 month; (2) Medical staff with practicing qualifications recognized by the National Health Commission and currently employed at the investigated hospitals; (3) Individuals capable of reading and understanding Chinese. The exclusion criteria were: (1) Participants with a confirmed history of mental illness; (2) Individuals who refused to participate.

### 2.2 Measures

#### 2.2.1 SWB

The revised version of the General Wellbeing Schedule (GWBS) developed by Fazio (54) and adapted by Duan et al. (55) was used to measure SWB. The scale is divided into 6 dimensions: satisfaction and interest in life, concerns about health, energy status, happy or depressed mood, control over emotions and behavior, and degree of tension or relaxation. The higher the total score on the scale, the stronger the SWB (55). A total score of ≤48 is considered “low SWB,” 49–72 is “moderate SWB,” and ≥ 73 is “high SWB” (56). The Cronbach’s α was 0.856.

#### 2.2.2 Burnout

The 22-item Maslach Burnout Inventory-Human Services Survey (MBI-HSS) (57) was used to measure burnout, which was divided into 3 dimensions: emotional exhaustion (EE), depersonalization (DP), and personal achievement (PA). The Chinese version was translated by Li et al. (58) and has good reliability and validity. The higher the score of EE and DP, the more serious the burnout; the higher the score of PA, the lower the degree of burnout. If the score of any dimension reaches or exceeds the threshold, the subject will detect burnout (59). The critical value of each dimension is defined as EE: low ≤16; moderate 17–26; high ≥27; DP: low ≤6; moderate 7–12; high ≥13; PA: low ≥39; moderate 32–38; high ≤31 (60). In this study, the Cronbach’s α was 0.781.

#### 2.2.3 PsyCap

PsyCap was measured using the Psychological Capital Questionnaire (PCQ-24) compiled by Luthans et al. (36). This questionnaire contains 4 dimensions of self-efficacy, hope, resilience, and optimism. Higher scores indicate higher levels of PsyCap. The Cronbach’s α of the total scale was 0.933.

#### 2.2.4 PSS

PSS was measured using the Perceived Social Support Scale (PSSS) developed by Zimet et al. (61) and revised by Qianjin (62). The scale includes perceived family, friends, and other support dimensions. The higher the score, the higher the PSS. The Cronbach’s α was 0.957.

### 2.3 Data analysis

All data were exported from the Questionnaire Star platform to SPSS (version 27.0, IBM Corp) for statistical analysis. All analyses were based on complete data. Before data analysis, Harman univariate tests were used to test for biases in common methods. Descriptive analysis (e.g., median, interquartile range (IQR), frequency, percentage) was used to present sample characteristics. Due to the non-normality of the data, Spearman correlation analysis was used to assess the relationship between variables. The SPSS macro PROCESS program (version 4.1) designed by Hayes (63) was used for mediation analysis using Model 6 to explore the relationship between burnout, PsyCap, PSS, and SWB. The Bootstrap confidence interval (CI) was set at 95%, with a Bootstrap sample size of 5,000. If the interval of the 95% CI did not include zero, it indicated a significant mediation effect. Type I error for all statistical analyses was *p* < 0.05 (two-sided).

### 2.4 Ethical considerations

This study was approved by the Ethics Committee of the School of Nursing, Jilin University (approval number: 2020030201). The survey was anonymous, confidential, and voluntary. Written informed consent was obtained from the participants before the survey (electronically, e.g., by clicking “yes”), and participants could choose to terminate the survey at any time.

## 3 Results

### 3.1 Common method deviation test

The data collected in this study comes from self-reports of the participants, so common method bias may exist. To reduce the impact of this bias on the study results, the Harman one-factor model method was used to test the degree of method bias in the data (64). The results showed that there were 12 factors with eigenvalues (i.e., the variances of the data in the directions of the eigenvectors) greater than 1, with the first factor explaining 29.56% of the variance, which was less than the critical standard of 40%, indicating that there was no serious homoscedasticity bias in the variables in this study (i.e., there was no serious variance attributable to the measurement method rather than the constructs of the measures in this study).

### 3.2 Demographic characteristics of participants

In this study, a total of 631 questionnaires were distributed. After eliminating 5 questionnaires with one or more missing answers and 22 questionnaires with 10 or more consecutively repeated responses, a total of 604 valid questionnaires were collected, achieving an effective response rate of 95.7%. The age of participants ranged from 19 to 64; 3.4% were physicians, 85.1% were nurses, and 1.5% were medical technologists (Table 1).

**Table 1:** Descriptive statistics of sample demographics and key variables (*n* = 604).

Variables	*n*	%
**Gender**		
Male	67	11.1
Female	537	88.9
**Age (years)**		
18–34	281	46.5
35–44	272	45.0
≥45	51	8.4
**Ethnicity**		
Han	544	90.1
Non-Han	60	9.9
**Education**		
Junior college and below	41	6.8
Bachelor	461	76.3
Master or above	102	16.9
**Marital status**		
Unmarried	126	20.9
Married	467	77.3
Divorced	11	1.8
**Children**		
None	185	30.6
One	351	58.1
Two or more	68	11.3
**Children’s stage**		
None	185	30.6
Infancy period	57	9.4
Pre-school age	134	22.2
School age	156	25.8
Puberty and above	72	11.9
**Employment type**		
Contract employment	335	55.5
Formal employment	269	44.5
**Working years**		
≤5	77	12.7
6–10	181	30.0
11–15	197	32.6
≥15	149	24.7
**Department**		
Surgery	88	14.6
Internal medicine	227	37.6
Emergency and ICU	115	19.0
Others	174	28.8
**Occupation category**		
Doctor	81	13.4
Nurse	514	85.1
Medical technologist	9	1.5
**Professional title**		
Junior	267	44.2
Intermediate	265	43.9
Senior	72	11.9
**Administrative position**		
No	566	93.7
Yes	38	6.3

ICU, intensive care unit.

### 3.3 Descriptive analysis of participants’ scale scores

Among the surveyed medical staff, the GWBS total score had a median of 78.00 (IQR: 68.00–89.00), with 1.8% reporting low SWB, 34.9% reporting moderate SWB, and 63.2% reporting high SWB. The lowest scored dimensions were “Satisfaction and interest in life” with a score of 7.00 (IQR: 6.00–8.00) and “Concerns about health” with a score of 6.00 (IQR: 5.00–8.00). The MBI-HSS total score had a median of 56.00 (IQR: 47.00–67.00), with a burnout detection rate of 52.5%, including 35.8% experiencing low burnout, 12.9% experiencing moderate burnout, and 3.8% experiencing high burnout. The PCQ total score had a median of 90.00 (IQR: 81.25–94.00), and the PSSS total score had a median of 65.00 (IQR: 54.00–72.00). The dimension scores of the scale are detailed in Table 2.

**Table 2:** Descriptive summary of subjective wellbeing, occupational burnout, psychological capital, and perceived social support (*n* = 604).

Scale/subscale	M	IQR	Degree		
			Low *n* (%)	Moderate *n* (%)	High *n* (%)
GWBS	78.00	68.00–89.00	11 (1.80)	211 (34.90)	382 (63.20)
Satisfaction and interest in life	7.00	6.00–8.00			
Concerns about health	6.00	5.00–8.00			
Energy status	19.00	16.00–22.00			
Happy or depressed mood	16.00	14.00–19.00			
Control over emotions and behavior	13.00	11.00–15.00			
Degree of tension or relaxation	16.00	14.00–20.00			
MBI-HSS	56.00	47.00–67.00	216 (35.80)	78 (12.90)	23 (3.80)
Emotional exhaustion	18.00	9.00–27.00	274 (45.40)	174 (28.80)	156 (25.80)
Depersonalization	3.00	0.00–6.00	484 (80.10)	62 (10.30)	58 (9.60)
Personal accomplishment	36.00	27.00–42.00	232 (38.40)	145 (24.00)	227 (37.60)
PCQ	90.00	81.25–94.00			
Self-efficacy	23.00	20.00–24.00			
Hope	23.00	20.00–24.00			
Resilience	22.00	20.00–24.00			
Optimism	21.00	19.00–23.00			
PSSS	65.00	54.00–72.00			
Family support	23.00	17.25–25.00			
Friend support	21.50	17.00–24.00			
Other support	21.00	17.00–24.00			

M, median; IQR, interquartile range; GWBS, General Wellbeing Schedule; MBI-HSS, Maslach Burnout Inventory-Human Services Survey; PCQ, Psychological Capital Questionnaire; PSSS, Perceived Social Support Scale.

### 3.4 Correlation analysis

The Spearman correlation analysis results indicated that burnout among medical staff was negatively correlated with SWB (*r* = −0.330, *p* < 0.01), PsyCap (*r* = −0.139, *p* < 0.01), and PSS (*r* = −0.127, *p* < 0.01); PsyCap was positively correlated with SWB (*r* = 0.642, *p* < 0.01) and PSS (*r* = 0.570, *p* < 0.01), thus meeting the requirements for intermediary effect analysis. Additionally, all correlation coefficients were below 0.700, indicating that there was no multicollinearity in the data (i.e., no high correlations between two or more variables) (see Table 3 for details).

**Table 3:** Correlation among variables (*r*, *n* = 604).

Variables	1	2	3	4
1. Subjective wellbeing	1.000			
2. Occupational burnout	−0.330^**^	1.000		
3. Psychological capital	0.642^**^	−0.139^**^	1.000	
4. Perceived social support	0.525^**^	−0.127^**^	0.570^**^	1.000

^**^P < 0.01 (two-tailed).

### 3.5 Mediation effect analyses

**Figure 2:**
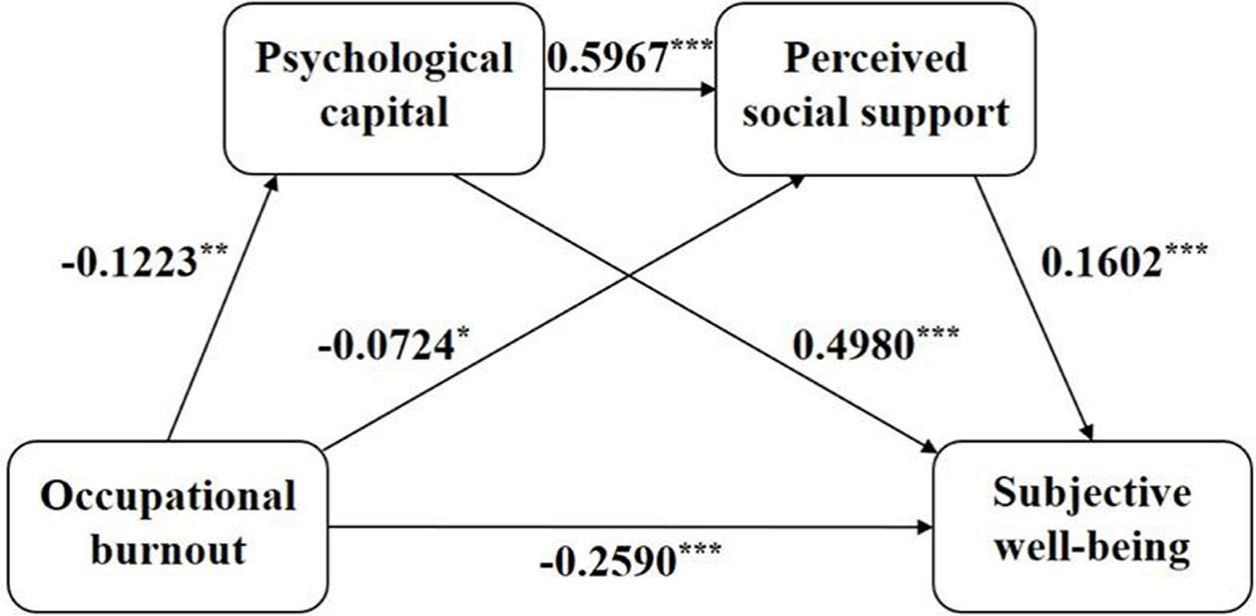
The chain mediating model of psychological capital and perceived social support on occupational burnout and subjective well-being. *P < 0.05, **P < 0.01, ***P < 0.001 (two-tailed).

As shown in Table 4 and Figure 2, firstly, burnout significantly negatively predicted the SWB of medical staff (*β* = −0.3432, *p* < 0.001, Model 1). Secondly, burnout had significant negative predictive effects on PsyCap (*β* = −0.1223, *p* < 0.01, Model 2) and PSS (*β* = −0.0724, *p* < 0.05, Model 3). Thirdly, PsyCap significantly positively predicted PSS (*β* = 0.5967, *p* < 0.001, Model 3). Fourthly, when burnout, PsyCap, and PSS simultaneously entered the regression equation (Model 4), the predictive effect of burnout remained significant (*β* = −0.2590, *p* < 0.001), and PsyCap (*β* = 0.4980, *p* < 0.001) and PSS (*β* = 0.1602, *p* < 0.001) both significantly positively predicted SWB.

**Table 4:** Regression analysis of the relationship between variables in the mediation effect model (*n* = 604).

Predictive variables	Model 1 (dependent variable: Subjective wellbeing)		Model 2 (dependent variable: Psychological capital)		Model 3 (dependent variable: Perceived social support)		Model 4 (dependent variable: Subjective wellbeing)	
	*β*	*t*	*β*	*t*	*β*	*t*	*β*	*t*
Occupational burnout	−0.3432	−8.9642^***^	−0.1223	−3.0231^**^	−0.0724	−2.2235^*^	−0.2590	−8.7031^***^
Psychological capital					0.5967	18.3191^***^	0.4980	13.4605^***^
Perceived social support							0.1602	4.3170^***^
*R*^2^	0.1178		0.0150		0.3719		0.4809	
*F*	80.3562^***^		9.1392^**^		177.9091^***^		185.3131^***^	

^*^P < 0.05, ^**^P < 0.01, ^***^P < 0.001 (two-tailed).

Finally, we used the Bootstrap method with percentile bias correction to test the mediating effects of PsyCap and PSS on the relationship between burnout and SWB. The results indicated that the mediating effects of PsyCap and PSS were significant, with the total indirect effect of 0.0665, accounting for 24.55% of the total effect (0.2709). Specifically, the mediating effect consisted of three paths supporting H1, H2, and H3. Firstly, the path coefficient of the indirect effect of burnout on SWB through PsyCap was −0.0481 (Bootstrap 95%CI: −0.0876, −0.0109). Secondly, the path coefficient of the indirect effect of burnout on SWB through PSS was −0.0092 (Bootstrap 95%CI: −0.0203, −0.0003). Thirdly, the path coefficient of the indirect effect of burnout on SWB through PsyCap and PSS was −0.0092 (Bootstrap 95%CI: −0.0183, −0.0019). Detailed results are available in Table 5 and Figure 2.

**Table 5:** Direct, indirect, and total effects of occupational burnout on subjective wellbeing (*n* = 604).

Effects	Pathways	Effect value	Bootstrap SE	Bootstrap 95% CI
Direct effect	Occupational burnout → Subjective wellbeing	−0.2045	0.0235	−0.2506, −0.1583
Indirect effects	Occupational burnout → Psychological capital → Subjective wellbeing	−0.0481	0.0196	−0.0876, −0.0109
	Occupational burnout → Perceived social support → Subjective wellbeing	−0.0092	0.0052	−0.0203, −0.0003
	Occupational burnout → Psychological capital → Perceived social support → Subjective wellbeing	−0.0092	0.0042	−0.0183, −0.0019
Total indirect effect		−0.0665	0.0237	−0.1126, −0.0199
Total effect		−0.2709	0.0302	−0.3303, −0.2116

SE, standard error; 95% CI, 95% confidence interval.

## 4 Discussion

In this study, we examined the relationship between medical staff’s burnout and SWB, as well as the mediating roles of PsyCap and PSS. Our findings indicate that PsyCap and PSS mediate the relationship between burnout and SWB. Therefore, the model suggests that burnout negatively influences PsyCap and PSS, leading to a decrease in SWB.

### 4.1 Current state of SWB of medical staff

In this study, the SWB of medical staff was slightly lower than that of the study results of Aggar et al. (65), indicating that the overall SWB of medical staff in China is slightly lower. The reason for this difference may be that their study participants were nurses in Australia, while China is a country with a large population base. With the opening of the two-child policy and the increasing aging population, the number of patients and the demand for medical care in tertiary public hospitals in China has reached an unprecedented level. There are only 6.94 professional public health personnel per 10,000 people, which is significantly lower than in other developed countries, causing each medical staff to provide services to more patients, requiring Chinese medical staff to work overtime frequently, with long working hours and heavy workloads (66). In addition, more than three-quarters of our research subjects are married, and the conflict between family and work may further lead to lower life satisfaction. Furthermore, the dimensions with the lowest scores are “Satisfaction and interest in life” and “Concerns about health,” which is similar to several studies (67–69), indicating that our study sample may experience a decrease in satisfaction and interest in life due to frequent shift work, disrupted circadian rhythms, and unresolved physical fatigue. At the same time, this study was conducted during the COVID-19 pandemic, during which medical staff frequently come into contact with highly infectious and critically ill patients, which may exacerbate their concerns about their health. A meta-analysis of the SWB of medical staff in China showed that the SWB of medical staff declined more significantly compared to other populations (e.g., college students, and older adults) (70).

### 4.2 The direct effect of burnout on SWB of medical staff

Wellbeing is broader than wellness and mainly focuses on personal responsibility (71). At present, research mainly measures the level of wellbeing in terms of SWB. SWB fluctuates throughout life (72) and is influenced by life events (73). This suggests that exploring potential pathways to improve the level of SWB is valuable, and understanding the factors and pathways that affect the level of SWB of medical staff is crucial for targeted interventions. Multiple studies have shown that burnout among medical staff is closely related to decreased SWB (25, 30, 49, 74). This indicates to healthcare system managers that the SWB of medical staff is not high and that their burnout is closely linked. It is important to pay attention to the relationship between the two and understand the factors and pathways that affect the level of SWB of medical staff to carry out targeted interventions. This study found that burnout significantly predicts a decrease in SWB among medical staff. This is consistent with previous findings in populations such as physicians, athletic coaches, and police officers (11, 75, 76), and this phenomenon may be related to the following factors. The Conservation of resources theory (77) posits that individuals have a natural inclination to seek and preserve valuable resources, such as time, wealth, optimism, and support. Whether a resource has been lost or is at risk of loss, it can cause stress to the individual (77). In the workplace, when individuals become aware that their resources are at risk and struggle to adjust to their jobs, it leads to a cycle of resource loss. This cycle creates job stress and eventually results in burnout (78). The progression of burnout amplifies an individual’s detrimental actions (e.g., heightened tobacco consumption, insufficient physical exercise, and lack of sleep), which subsequently results in various physiological manifestations; moreover, burnout tends to generate more frequent adverse emotions, giving rise to psychological symptoms like anxiety and depression (79). Burnout results in the exhaustion of both physical and mental resources. This exhaustion then makes it difficult for individuals to effectively allocate time and effort toward other responsibilities (e.g., family roles). It also creates conflicts and disruptions in their ability to balance their work and family life (80, 81). SWB is primarily comprised of cognitive and affective elements, which encompass evaluations of life satisfaction and positive/negative emotional reactions (9). Burnout can cause damage to physical and mental health, and work–family conflict can lead to a decrease in SWB. Current research provides supportive evidence for this hypothesis. The research results also inspire us to strengthen the improvement of burnout among medical staff, which is very necessary for enhancing SWB.

### 4.3 Mediation effects of PsyCap and PSS

This study demonstrates that PsyCap plays an important mediating role in the relationship between burnout and SWB among Chinese medical staff, confirming H1. This means that medical staff with high levels of burnout are more likely to have lower PsyCap, leading to a decrease in SWB. This is similar to previous findings in the nursing population (44, 82). The mechanisms may be related to the following factors: the Job Demands-Resources model (JD-R) suggests that the interaction between job demands and job resources affects the development of burnout (83). Job resources, which include a combination of internal and external resources, can reduce the impact of job demands on burnout (84). As an important internal positive psychological resource, individuals with high levels of PsyCap generally have a positive attitude and confidence in their work, and offer more positive interpretations of work events, making it easier for them to recover from setbacks and failures (85, 86). Additionally, the broaden hypothesis in the Broaden-and-Build theory proposes that positive emotions increase an individual’s focus and awareness of their environment, allowing them to have a broader range of thoughts and perceptions than normal (87). This enhances wellbeing due to increased physical, psychological, and social resources (87). In this sense, PsyCap gives individuals the ability to innovate, etc. (88, 89), which improves the SWB in terms of individual psychological ability and strength. In addition, those with high levels of PsyCap are better able to overcome stress appropriately, feel more comfortable physically and mentally, and tend to have positive outcomes on SWB (90). This is similar to a cross-sectional study result conducted by Ravikumar among medical staff and police personnel during the COVID-19 pandemic (91). They found that positive PsyCap helps reduce occupational stress and improve mental health. This finding suggests that despite the negative impact of burnout on SWB, medical staff with sufficient PsyCap resources can mitigate this impact and reduce the damage to SWB.

This study also found that the PSS is an intermediate variable between burnout and SWB among Chinese medical staff, confirming H2. This is consistent with previous research findings (92). The underlying mechanism may be related to the following factors: firstly, the PSS as an important external resource may improve burnout by reducing job demands and supporting the JD-R model perspective from another angle (84). When individuals receive support from their work group, external work resources increase, effectively reducing the occurrence of burnout. Additionally, previous research has shown that work relationships characterized by lack of support and trust increase the risk of burnout (93). Conversely, when work relationships are positive and there is a high level of social support, employees are more likely to resolve conflicts effectively and be more engaged in their work. For medical staff, perceiving support from family, friends, or others can reduce negative emotions such as fatigue or indifference, increase personal achievement or confidence, and thereby reduce the occurrence and development of burnout. A narrative review highlighted that during the COVID-19 pandemic, peer support as one of the sources of social support, is a key factor in managing work stress (51). Furthermore, the theory of subjective wellbeing homeostasis indicates that SWB is maintained by stable forces such as adaptation and positive emotions (94). At the same time, the maintenance of SWB is influenced by emotional responses, and if internal stable resources are strong, SWB will remain within a normal range (94). As a positive social psychological resource for individuals, PSS can help buffer stress sources in the body’s balancing system, thereby maintaining the relative stability of an individual’s SWB. Therefore, for medical staff, PSS as a buffer for burnout can help individuals cope with burnout, reduce the negative effects of burnout on physical and mental health, and thus affect SWB.

In addition, this study provides small but significant support for the chain intermediary role of PsyCap and PSS between burnout and SWB among medical staff, which has not been explored in previous studies. In the mediation path of burnout and SWB, PsyCap plays a partial mediating role in the relationship between burnout and PSS, indicating that positive PsyCap can serve as an important buffer to prevent the impact of burnout on the PSS among medical staff. Positive emotions can lead individuals to a growth trajectory, enabling them to identify and build their resources, and these resources are enduring (87). As an inherent positive psychological resource within individuals, multiple studies (95, 96) have confirmed that individuals with high levels of PsyCap generally perceive more prominent material and emotional support between family members and peers, which provides support for the build hypothesis in the broaden-and-build theory. Furthermore, the burnout experienced by medical staff can lead to a decrease in positive psychological resources such as low hope levels and low self-efficacy. At the same time, psychological resources serve as an important mediator for PSS. Faced with the vast work system and personnel structure of tertiary hospitals, individuals with low psychological resources are more likely to show social detachment from colleagues and experience a decrease in PSS, which in turn leads to lower SWB. Therefore, burnout can affect the SWB among medical staff through the chain intermediary role of PsyCap and PSS.

Existing intervention research has found that positive psychology interventions can enhance the PsyCap level of primary care physicians, change work behavior (e.g., coping with work more effectively), improve work-related outcomes (e.g., burnout), and thus influence wellbeing (97). Moreover, interventions including a module that enhances PSS can improve certain components of wellbeing, particularly work stress (98). This result inspires us to develop positive psychology interventions or to develop psychological interventions that include enhancing PsyCap and PSS components, to improve medical staff’s burnout and promote SWB.

### 4.4 Relevance to clinical practice

In China, even if they do not have serious illnesses, patients tend to prefer seeking medical treatment at large public hospitals (99). This results in a high outpatient volume and a large number of hospitalized patients at tertiary hospitals, which can easily lead to burnout among medical staff (100), but the clinical demand for healthcare services is increasing. Therefore, it has become particularly important to improve the SWB of medical staff who are the main body for saving lives. This study has certain theoretical significance and practical value for improving the SWB of medical staff. Given the important mediating role of PsyCap and PSS in the relationship between burnout and SWB, we propose the following suggestions to improve medical staff burnout and ultimately enhance their level of SWB. Firstly, it is important to focus on and enhance individuals’ level of PsyCap. Luthans et al. (101) argue that PsyCap is a state-like individual resource that can be changed and improved. The methods proposed by Luthans et al. (35, 101) for developing PsyCap include: allowing employees to experience success to develop self-efficacy and confidence; accepting their past mistakes, failures, and setbacks; encouraging them to appreciate the present and be grateful for positive aspects of life, etc. Secondly, it is important to prioritize and enhance the individual’s PSS as much as possible. This is closely related to the level of individual PsyCap and the harmonious organizational atmosphere. Therefore, we hope to strengthen the attention to burnout and wellbeing among medical staff at the policy-making level, create a positive work atmosphere at the organizational leadership level, and focus on enhancing positive psychological resources at the individual level.

### 4.5 Limitations

This study also has notable limitations. Firstly, it is a cross-sectional survey design, which cannot establish a causal model. Therefore, further investigation and validation should be conducted by combining longitudinal studies and *in vitro* and *in vivo* experiments to further uncover the specific mechanisms of the mediating effect in this study. Secondly, the data is sourced from a sample of medical staff in Changchun, Jilin Province, China, which limits the generalizability of the research findings. Lastly, the assessment of variables is based on self-reported results, which cannot exclude response bias.

## 5 Conclusion

Based on determining the current status of medical staff’s burnout and SWB, this study constructed a mediation model to investigate the processes and mechanisms by which burnout affects medical staff’s SWB. The research findings indicate that high levels of burnout not only lead to a decrease in the SWB of medical staff but also affect their PsyCap and PSS. Additionally, burnout decreases the PsyCap and PSS among medical staff, thereby reducing their SWB. Furthermore, a novel contribution of this current study is the exploration of PsyCap and PSS as chain intermediary roles in burnout and SWB. This study provides initial data support for the design of intervention strategies targeting medical staff burnout and SWB. Enhancing PsyCap and PSS are potential strategies for reducing burnout and improving SWB. Hospital managers should take corresponding measures to enhance medical staff’s PsyCap and PSS, thereby improving their SWB. This will play a certain role in promoting the quality and level of medical staff’s services, while ensuring the vigorous and stable development of the national healthcare system. In addition, this will provide hospital administrators and medical staff with better reference for coping with potential sudden public health emergencies in the future.

## Data availability statement

The raw data supporting the conclusions of this article will be made available by the authors, without undue reservation.

## Ethics statement

The studies involving humans were approved by Ethics Committee of the School of Nursing, Jilin University (approval number: 2020030201). The studies were conducted in accordance with the local legislation and institutional requirements. The participants provided their written informed consent to participate in this study.

## Author contributions

JF: Conceptualization, Methodology, Writing – original draft. YC: Conceptualization, Formal analysis, Investigation, Writing – original draft. LL: Data curation, Investigation, Writing – original draft. NJ: Investigation, Resources, Writing – original draft. ZQ: Data curation, Resources, Writing – original draft. JZ: Formal analysis, Resources, Writing – original draft. ML: Formal analysis, Visualization, Writing – original draft. BL: Supervision, Writing – review & editing. DQ: Supervision, Writing – review & editing.

## Footnotes

^1^ https://www.wjx.cn/
